# Isolated Pyomyositis of the Thigh With a Sinus Tract: A Rare Presentation of Tuberculosis

**DOI:** 10.7759/cureus.58788

**Published:** 2024-04-22

**Authors:** Saumya Singh, Gunjan Parasher, Vaibhav Jaiswal, Jyoti Bajpai, Shailendra Kumar

**Affiliations:** 1 Department of General Surgery, King George's Medical University, Lucknow, IND; 2 Department of Trauma and Acute Care Surgery, King George's Medical University, Lucknow, IND; 3 Department of Respiratory Medicine, King George's Medical University, Lucknow, IND; 4 Department of Surgery: Thoracic Surgery, King George's Medical University, Lucknow, IND

**Keywords:** percutaneus catheter drainage, mycobacterium tuberculous, anti tubercular therapy (att), sinus tract, extrapulmonary tuberculosis (eptb), pyomyositis

## Abstract

Muscular tuberculosis as a primary focal lesion in an immunocompetent individual without any underlying bone involvement is a rare finding. The authors present a case of a young female in her 30s who presented with complaints of recurrent discharging sinus in the posteromedial aspect of the proximal right thigh for eight months. The patient was treated by surgical debridement followed by antitubercular therapy (ATT) and has shown full recovery during the post-eight-month treatment period. Such a presentation of primary tubercular pyomyositis imposes a diagnostic as well as a therapeutic challenge.

## Introduction

Extrapulmonary tuberculosis (EPTB) may present as lymphadenitis (often cervical), pleuritis, meningitis, abdominal TB (including peritonitis), skeletal TB, such as Pott disease (spine), and genitourinary (renal) TB. Extrapulmonary infection accounts for 20%-40% of all tuberculosis cases [1]. Extrapulmonary TB is difficult to diagnose because of its nonspecific manifestation, which leads to a delay in its diagnosis. Tuberculosis pyomyositis is a rare entity, constituting less than 1% of all cases of skeletal tuberculosis [2]. This can mimic inflammatory myositis or, rarely, neoplasia of the region and can create diagnostic confusion for the surgeons. Isolated muscular tuberculosis in the extremities of immunocompetent individuals is a rare affliction and seldom reported in the medical literature. Further, on a wide search done on PubMed, we found few such cases reported from the year 2001 until now. The absence of any clinical or serological evidence of immunodeficiency and no evidence of pulmonary tuberculosis or previous anti-tubercular therapy (ATT) complicates the diagnosis. Extrapulmonary tuberculosis (EPTB) resulting from Mycobacterium tuberculosis (MTb) responds to first-line anti-TB medicines. Certain cases of EPTB have also been treated with adjuvant therapy consisting of corticosteroids in addition to antibiotics. Surgical intervention is sometimes advised, primarily when the patient's organ damage is incapacitating. To treat drug-resistant EPTB cases (DR-EPTB), a drug susceptibility profile is a crucial requirement. In an immunocompetent woman, we present a case of tubercular pyomyositis of the hamstring group of muscles.

## Case presentation

**Figure 1:**
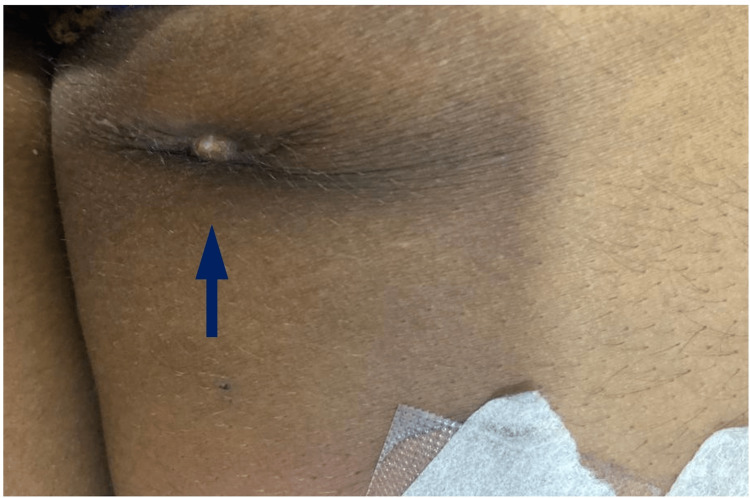
A blind sinus tract is present at the posteromedial aspect of the right thigh.

We report a case of a young adult female who presented to our tertiary care medical center with complaints of recurrent spontaneous yellowish purulent discharge from the posteromedial aspect of the right thigh for the last eight months. The patient received antibiotics from a local practitioner for swelling in the thigh that remained asymptomatic for three months but then ruptured spontaneously, exuding purulent discharge, and then the patient was referred to us. There was no history of fever, weight loss, anorexia, or other constitutional symptoms of TB. There was no preceding history of trauma, injection at the concerned site, muscular weakness, fever, weight loss, diabetes, renal disorder, contact with TB, ATT intake, steroid intake, or previous surgery. We found a healed, indurated, non-tender scar (2 x 2 cm) on the posteromedial aspect of the right thigh (Figure 1).

**Figure 2:**
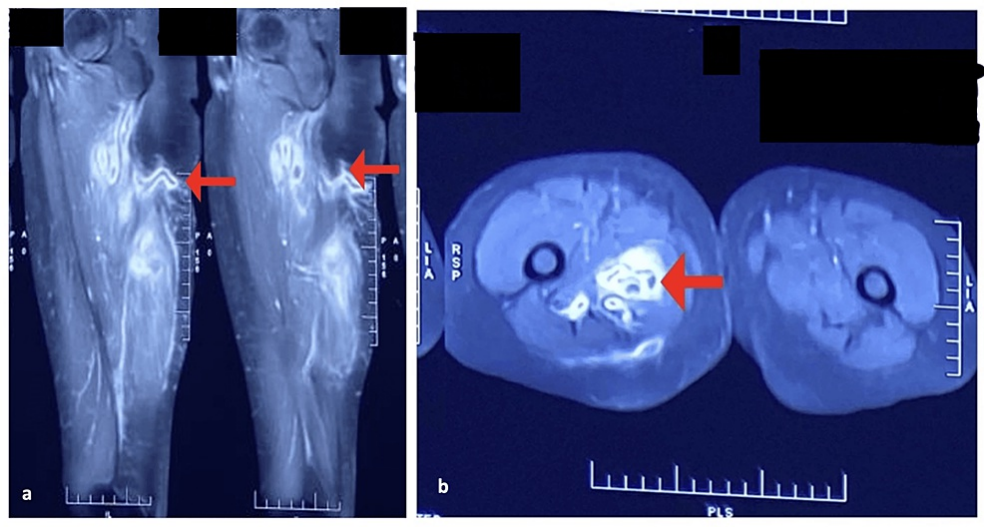
CEMRI results. A: CEMRI sagittal view, showing a peripherally enhanced L-shaped sinus tract with an external opening in the posterior aspect of the proximal thigh; B: CEMRI axial view, T1 and T2W images showing a peripherally enhanced L-shaped sinus tract with an external opening in the posterior aspect of the proximal thigh. CEMRI: contrast-enhanced magnetic resonance imaging

The hemogram, liver, and kidney function tests of the patient were normal, along with typical glycated hemoglobin (HbA1c) values, and the patient was negative for the human immunodeficiency virus (HIV) and hepatitis B and C viruses (HBV and HCV). The chest X-ray was clear. A sinogram of the right thigh suggested a long tract and contrast-enhanced magnetic resonance imaging (CEMRI) revealed a peripherally enhancing L-shaped sinus tract, measuring approximately 5.3 centimeters in length with an external opening in the posterior aspect of the proximal thigh (Figure 2).

The tract traversed anteriorly and medially through the subcutaneous plane, piercing the adductor magnus and forming an inter-communicating abscess (31 x 30 x 171 mm) within the hamstring group of muscles. No evidence of skeletal or neurovascular involvement was found. Other groups of muscle and connective tissues were normal. The histopathology report suggested the tubercular origin of the lesion by revealing acid-fast bacilli. Since no other foci of infection were found elsewhere in the body and no history of constitutional symptoms of tuberculosis was evident, we concluded the final diagnosis as primary tubercular pyomyositis.

The differentials considered in the patient included infective pathology: bacterial or fungal abscess, inflammatory or degenerative disorders, infected Cysticercus cellulose (parasitic infections), focal myositis, and soft tissue tumors like fibroma, hemangioma, and myxoma, which were ruled out based on radiological and microbiological evidence.

**Figure 3:**
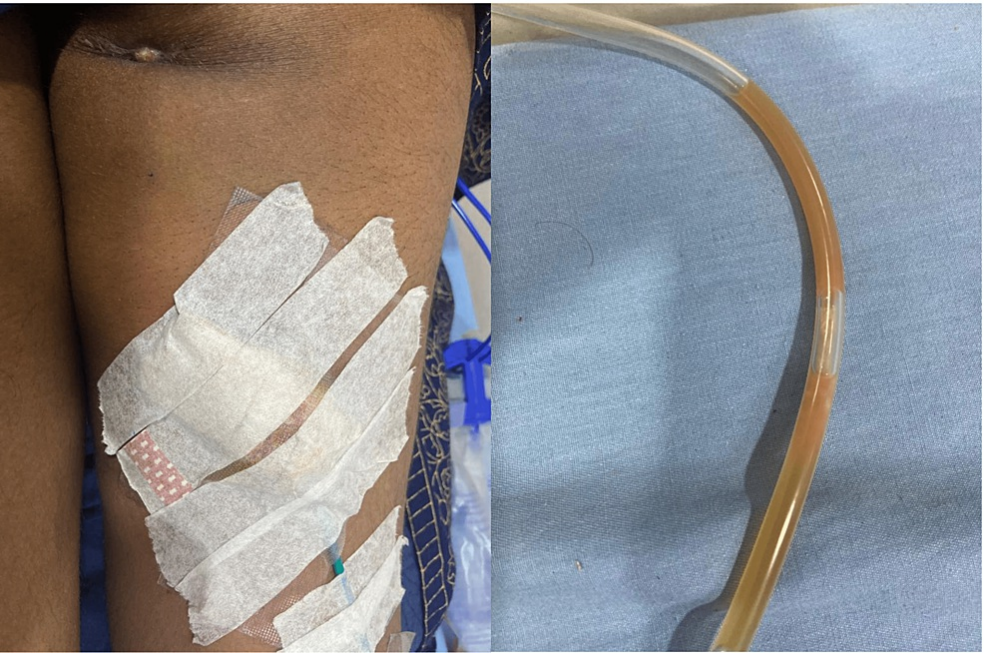
Catheter in situ (a) and purulent discharge in the catheter (b).

The intramuscular abscess was drained via an ultrasound-guided percutaneous catheter for two weeks (Figure 3).

Smears from the collected pus showed acid-fast bacilli, indicating the tubercular origin of the abscess. A molecular test called the cartridge-based nucleic acid amplification test (CBNAAT) was done and showed acid-fast bacilli (AFB) positive, but rifampicin resistance was absent. Culture was also done, and it came out positive but sensitive to first-line drugs. The patient was referred to the Department of Pulmonary Medicine for antitubercular drug sensitivity evaluation and the best course of ATT. The strain was susceptible to all the first-line antitubercular drugs. The patient was started on the intensive phase of therapy with weight-adjusted doses of isoniazid, rifampicin, pyrazinamide, and ethambutol.

The patient was followed up on a four-weekly basis. At the end of the intensive phase (two months) of ATT, the patient reported no complaints about discharge. The therapy was continued for another four months. On follow-up, after eight months, the patient was asymptomatic and fully recovered. Follow-up imaging and high-resolution ultrasound showed no residual collection with minimal fibrosis in the subcutaneous plane.

## Discussion

Pyomyositis is the term used to describe a bacterial infection of the skeletal muscles with the formation of an abscess within them. The exact etiology is still unknown, but factors like trauma, nutritional deficiencies, viral infections, septic load, and parasitic infestations have been implicated as predisposing factors [3]. Infection by Mycobacterium tuberculosis is an infrequent cause of pyomyositis. This is mainly because the striated muscles are most resistant to the bacteria because of their poor oxygen content, high lactic acid content, and scarcity of reticuloendothelial tissue [4]. The delay in diagnosis is due to a lack of awareness about this disease, unfamiliarity with this entity, atypical presentations, a lack of early specific signs, and a wide range of differential diagnoses. Because of the late presentation and treatment, a large group of muscle fibers get affected, resulting in atrophy and contractures [5].

Tuberculous abscesses are predominantly seen in the thoracic and abdominal wall, followed by the paravertebral line and lymph nodes, and rarely is the involvement of subcutaneous and muscle tissues in limbs seen [6]. Muscular involvement of extremities by tuberculosis is attributed to direct inoculation by underlying bone and rarely by hematogenous dissemination in immunocompromised individuals [7]. The pathogenesis of such a presentation remains unclear.

A case was reported in 2011 of tuberculous pyomyositis in the thigh in an immunocompromised patient [8]. Puttick MP et al. reported a case series of 11 patients with soft tissue tuberculosis. Six of them had collagen vascular disorders, one had a kidney transplant, and five were on immunosuppressive therapy and/or prednisone. Three had previous trauma to the affected area. Five of them had evidence of previous tuberculosis in their histories or chest radiographs [9]. Additionally, it was observed in the few reported cases that no history of constitutional symptoms of tuberculosis was found, indicating the indolent nature of the disease [10,11]. In our case, the patient neither had concurrent active tuberculosis (local/distant) nor any history of tuberculosis. This might further pose a dilemma for the physicians to suspect Mycobacterium as the potential pathogenic agent. Most of the cases are initially suspected and treated as bacterial pyomyositis; thus, diagnosis requires a high degree of suspicion, especially in tuberculosis-endemic countries [11]. Many cases of tubercular pyomyositis have been reported in the literature, most of which were seen to be limited to one muscle [9,12,13]. In our case, there was a group of muscles involved, which included the long and short heads of the biceps femoris, semitendinosus, and semimembranosus. Blood investigations are usually normal, as is in our case [4]. Radiological imaging may help to comprehend the extent of involvement, but histopathology, along with culture, is the only means to establish a definitive diagnosis. Newer molecular tests like CBNAAT and line probe assay (LPA) are also instrumental in the diagnosis of extrapulmonary samples. Substantial delay was noted in the disease diagnosis, ranging from two months to two years in various cases [14]. MRI is the investigation of choice. In a case study in which the MRIs of four cases of tuberculous pyomyositis were retrospectively analyzed with signal intensity for the presence of an abscess, it was found that all cases showed characteristic findings of well-demarcated abscesses and could be distinguished from the other soft tissue masses [4]. In our case, MRI showed a formation of collection-abscess (approximately 31 x 30 x 171 mm) within the hamstring group of muscles, with peripheral short tau inversion recovery (STIR) hyperintensity suggestive of edema noted in the periphery of the abscess and no evidence of any communication with the intramedullary cavity. Femoral vessels were away from the abscess, which showed there was no hematogenous spread. In a tubercular-endemic country like India, we present this case of primary tubercular pyomyositis in an immunocompetent patient without any identifiable focus elsewhere in the body. The prognosis would be good with a multimodal approach, including drainage of the abscess, surgery for pyomyositis, debridement of devitalized tissue, and antitubercular treatment.

## Conclusions

Primary muscular tuberculosis may present in endemic regions without any discernible constitutional features in immunocompetent individuals. A CBNAAT or True NAT as the first-line test should be done before starting treatment if an infectious cause is suspected to reduce chronicity and prevent complications. Samples like pus and tissues should be sent for smear microscopy, molecular tests, CBNAAT, line probe assay (LPA), histopathology, and culture to diagnose extrapulmonary tuberculosis. Magnetic resonance imaging with gadolinium enhancement helps diagnose intramuscular abscesses. The disease prognosis is good, primarily due to the non-involvement of bony structures and Mycobacterium being fully responsive to the available antitubercular drugs. These unusual scenarios require further analysis to understand the pathogenesis and deliver treatment per standard of care.

## References

[1] Wang WY, Lin FC, Tsao T, Lu J (2007). Tuberculous myositis: an unusual presentation of extrapulmonary tuberculosis. Journal of Microbiology, Immunology and Infection.

[2] Abdelwahab IF, Bianchi S, Martinoli C, Klein M, Hermann G (2006). Atypical extraspinal musculoskeletal tuberculosis in immunocompetent patients: part II, tuberculous myositis, tuberculous bursitis, and tuberculous tenosynovitis. Canadian Association of Radiologists Journal.

[3] Chauhan S, Jain S, Varma S, Chauhan SS (2004). Tropical pyomyositis (myositis tropicans):current perspective. Postgraduate Medical Journal.

[4] Kim JY, Park YH, Choi KH, Park SH, Lee HY (1999). MRI of tuberculous pyomyositis. J Comput Assist Tomogr.

[5] Sen RK, Tripathy SK, Dhatt S, Saini R, Aggarwal S, Agarwal A (2010). Primary tuberculous pyomyositis of forearm muscles. Indian Journal of Tuberculosis.

[6] Gao W, Zeng Y, Chen W (2020). Multiple subcutaneous tuberculous abscesses in a dermatomyositis patient without pulmonary tuberculosis: a case report and literature review. BMC Infect Dis.

[7] Sökücü S, Sökücü SN, Kabukçuoglu Y, Kabukçuoglu F (2013). Primary skeletal muscle tuberculosis at an unusual site. J Pak Med Assoc.

[8] Puttick MP, Stein HB, Chan RM, Elwood RK, How AR, Reid GD (1995). Soft tissue tuberculosis: a series of 11 cases. J Rheumatol.

[9] Hasan N, Baithun S, Swash M, Wagg A (1993). Tuberculosis of striated muscle. Muscle and Nerve.

[10] Kumar U, Dasgupta S (2019). Musculoskeletal tuberculosis: a multifaceted foe. Ann Natl Acad Med Sci.

[11] Modi MA, Mate AD, Nasta AM, Gvalani AK (2013). Primary tuberculous pyomyositis of quadriceps femoris in an immunocompetent individual. Case Reports in Infectious Diseases.

[12] Chen WS (1998). Tuberculosis of the fascia lata. Clinical Rheumatology.

[13] Sridhar C, Seith A (2004). Tuberculous pyomyositis of the temporalis muscle. European Journal of Radiology Extra.

[14] Zhu XW, Luan XH, Jiang KL (2023). Case report: muscular tuberculosis with lower-extremity muscular masses as the initial presentation: clinicopathological analysis of two cases and review of the literature. Front Med (Lausanne.

